# Regulation Mechanism of Different Metal Cations on the Structure and Gel Properties of Montmorillonite

**DOI:** 10.3390/ma18081878

**Published:** 2025-04-20

**Authors:** Sixiao Wang, Dinghua Liu, Tiantian Zhang, Haowei Yan, Zepeng Zhang, Junming Geng

**Affiliations:** 1 Engineering Research Center of Ministry of Education for Geological Carbon Storage and Low Carbon Utilization of Resources, Beijing Key Laboratory of Materials Utilization of Nonmetallic Minerals and Solid Wastes, National Laboratory of Mineral Materials, School of Materials Science and Technology, China University of Geosciences (Beijing), Xueyuan Road, Haidian District, Beijing 100083, China; wangsx1009@163.com (S.W.); tiantianzhangzzz@163.com (T.Z.); yhwcugb2019@gmail.com (H.Y.); 2 Suzhou Guojian Huitou Mineral New Material Co., Ltd., Suzhou 215151, China; 3 Suzhou Sinoma Design and Research Institute of Non-Metallic Minerals Industry Co., Ltd., Suzhou 215151, China; 15650700916@163.com; 4 School of Chemistry and Chemical Engineering, Beijing Institute of Technology, Beijing 102488, China

**Keywords:** montmorillonite, different metal cations, regulation mechanism, structure of montmorillonite, gel properties of montmorillonite

## Abstract

Metal cations are often used to regulate montmorillonite, but the mechanism is still unclear. In this paper, the regulation of different cations in montmorillonite was studied, and it was found that the regulation of different cations had significant effects on the structure of montmorillonite. Firstly, the viscosity is negatively correlated with particle size, and the order of particle size is trivalent > divalent > monovalent cation. Secondly, the swelling capacity is positively correlated with the absolute value of zeta potential, and the order of the zeta potential is monovalent > trivalent > divalent cation. Thirdly, the smaller hydrated ion radius and static electricity of monovalent cations significantly reduce the layer spacing. Meanwhile, isomorphism displacement results in a significant increase in the proportion of cis-vacant configuration due to changing the electronegativity of the octahedron. The comprehensive performance is that the particle size is significantly reduced and the absolute value of zeta potential is significantly increased. It is easy to peel off and expand in water to form a uniform and stable colloidal substance, which has the best gel performance. The research results can provide theoretical support for the regulation of montmorillonite structure and gel properties by different valence metal cations.

## 1. Introduction

Montmorillonite (Mt), a typical layered silicate clay mineral, is widely utilized owing to its abundant reserves, relatively low cost, and remarkable adsorption, suspension, and thermal stability, thus earning the title of “universal clay”. The gel property is among the most crucial characteristics of montmorillonite [1].

Isomorphic substitution readily takes place in the crystal structure of montmorillonite, leading to a high local charge density on the surface of the montmorillonite flakes. Consequently, the montmorillonite flakes display strong hydrophilicity [2,3,4]. When montmorillonite is mixed with water, the water permeates into the sheets, prompting the montmorillonite layer to expand and exfoliate, and exchangeable ions to diffuse from the surface of the sheets. Due to the osmotic effect, water molecules further infiltrate between the sheets until the sheets are completely separated. For these dispersed montmorillonite sheets, the edges of the weakly positively charged sheets are attracted to the negatively charged sheet surface, and a “cardboard” three-dimensional spatial structure is formed via electrostatic interaction, thereby creating a gel system [5].

Over the past decade, predecessors have conducted extensive research on the properties of montmorillonite gels. Gayle E. Morris et al. [6] employed acoustics and advanced low-temperature scanning electron microscopy to investigate the structural changes and stability of montmorillonite aqueous suspensions in the presence and absence of calcium ions. It was discovered that Ca^2+^ exerts a substantial influence on the stability and structure of montmorillonite suspensions. Jun Qiu [7] explored the relationship between the crystal chemical properties of montmorillonite with varying layer charge densities and its dispersion properties. The study revealed that the charge density was positively correlated with the mass content of MgO. Moreover, the results of Fourier transform infrared spectroscopy (FTIR) and scanning electron microscopy (SEM) analyses indicated that the dispersion of montmorillonite decreased as the layer charge density increased. Yan Haowei et al. [1] examined the effect of metal ions on the gel properties of montmorillonite and found that montmorillonite with low-valence interlayer cations had a larger absolute value of the zeta potential and better gel properties. Additionally, among cationic montmorillonite with the same valence state, those with a larger ionic radius exhibited better gel properties. Sun Hongjuan et al. [8] utilized multiple metal cations to modify purified montmorillonite and prepared a series of montmorillonite samples such as those containing Li, Na, K, Mg, Ca, and Ba under the same experimental conditions. These samples were analyzed through X-ray diffraction (XRD), ultraviolet–visible (UV–Vis) absorption spectroscopy, and swelling capacity tests. The study demonstrated that when the montmorillonite layer contained a divalent cation, the number of water molecular layers was two, whereas when the interlayer had a monovalent cation, the number of water molecular layers was one. Haowei Yan et al. [1] investigated the gel properties of montmorillonite substituted by different valence metal cations. The results indicated that the electrical properties and interlayer forces of montmorillonite platelets were affected by the valence state of metal cations, electrostatic forces, and the radius of hydrated ions, which in turn influenced the gel properties of montmorillonite. Xiaoli Wang et al. [9] characterized the structure of the octahedral sheet in dioctahedral montmorillonite via thermal analysis. The results showed that the iron content had a significant impact on the structure of the octahedral sheet.

It is evident that, firstly, the majority of current research focuses on regulating the structure of montmorillonite using a single cation to explore the alterations in its gel properties, while lacking an in-depth analysis of the distinct regulatory mechanisms of different valence metal cations on the montmorillonite structure. Secondly, although researchers have conducted modification experiments on purified montmorillonite with diverse metal cations to elucidate the variation patterns of its structure, the connection between the changes in montmorillonite structure and its gel properties remains unestablished. Thirdly, although relevant studies have reported the influence mechanism of the interlayer structure variation on the gel properties of montmorillonite, the correlation between the isomorphism of different ion substitutions within the montmorillonite crystal structure and its gel properties is still absent.

In this study, a systematic analysis was conducted on the relationship between the basic properties of montmorillonite and its gel properties. Regulation experiments involving different valence metal cations were performed on selected samples. Subsequently, the interlayer structure of montmorillonite samples regulated by different valence metal cations was elucidated through X-ray diffraction (XRD) and low-temperature differential thermal gravimetry (DTG) analysis within the temperature range of 50–300 °C. Additionally, the crystal structure of the montmorillonite samples regulated by different valence metal cations was investigated by inductively coupled plasma (ICP) and 27Al cross-polarized magic angle spinning nuclear magnetic resonance (CP-MAS). Notably, the cv configuration ratio of the octahedral structure of montmorillonite was quantified by fitting the DTG curve in the temperature range of 300–800 °C. Finally, the regularity of different valence metal cations in regulating the interlayer and crystal structures of montmorillonite was summarized, and the mechanism underlying the changes in montmorillonite structure and gel properties such as viscosity and swelling capacity was analyzed. The research findings offer theoretical support for the regulation of montmorillonite structure and gel properties by different valence metal cations.

## 2. Materials and Methods

### 2.1. Materials and Reagents

The five kinds of raw montmorillonite ore selected in this research are from Liaoning, Hebei, and Inner Mongolia of China, as shown in Table 1. All five samples were purified by natural sedimentation and were marked as Mt1, Mt2, Mt3, Mt4, and Mt5, respectively.

Sodium carbonate was purchased from Tianjin Bodi Technology Development Co., Ltd. (Tianjin, China), lithium carbonate from Shanghai Aladdin Biochemical Technology Co., Ltd. (Shangai, China), magnesium carbonate basic pentahydrate from Xilong Scientific Co., Ltd. (Shantou, China), zinc chloride from Tianjin Hengxing Chemical Reagent Manufacturing Co., Ltd. (Tianjin, China), aluminum chloride hexahydrate from Shanghai Runjie Chemical Reagent Co., Ltd. (Shanghai, China), and ferric chloride from McLean Biochemical Technology Co., Ltd. (Shanghai, China). All of the reagents listed above were of analytical grade.

### 2.2. Characterization

#### 2.2.1. XRD

XRD was measured using a D8 Advance X-ray diffractometer produced by Bruker Scientific Instruments Hong Kong. The diffractometer was equipped with a Cu source with a wavelength of 1.540601 Å, operating voltage of 60 kV, with a step size of 0.02°, and the data were collected in the 2θ range 3–70°. All samples were dried at 60 °C for 24 h prior to the XRD analysis.

According to the Scherrer formula, Equation (1), the thickness of montmorillonite grains in the 001 crystal plane direction had been calculated.

(1) $$D_{001}=\frac{K\lambda }{\beta cos\theta }$$

where D_001_ is the thickness of montmorillonite grains in the 001 crystal plane direction (nm), K is the Scherrer constant, generally taken as 0.89, λ is the X-ray wavelength (0.1541 nm), β is the half-width (radian) of the 001 diffraction peak, and θ is the 001 diffraction angle (°).

#### 2.2.2. Particle Size Distribution (PSD)

The montmorillonite samples that had been dried at 60 °C for 24 h were added into deionized water to prepare a suspension with a mass fraction of 1%. The JL-1177 laser particle size distribution tester was manufactured by Chengdu Jingxin Powder Testing Equipment Co., Ltd. (Chengdu, China).

#### 2.2.3. Zeta Potential

Weigh each sample 0.01 g, add 10 mL of distilled water, respectively, and ultrasonic oscillation for 10 min to obtain the test sample. Zetasizer Nano ZS90 and Zeta potentiometer of Malvern Instruments Co., Ltd. (Malvern, UK) were used to test the samples, and the test temperature was 25 °C. Each sample was tested 3 times; the relative error was less than 0.5%.

#### 2.2.4. Thermogravimetric Analysis

Thermogravimetric (TG) analysis was carried out using the STA 449C thermal analyzer from German Netzsch (Selb, Germany). The test temperature ranged from room temperature to 1000 °C, with a heating rate of 10 °C/min. A nitrogen atmosphere was utilized during the analysis.

#### 2.2.5. ICP

For each viscosity test sample, 25 mL was centrifuged to obtain the supernatant. An amount of 25 mL of deionized water was added and this process was repeated for 4 times. Subsequently, the supernatant was adjusted to a volume of 100 mL. Then, each sample was filtered through a polyethersulfone filtration membrane with a pore size of 0.22 μm to obtain the sample for inductively coupled plasma (ICP) testing. Each sample was characterized using the Agilent 7500, an inductively coupled plasma emission spectrometer provided by Agilent Technologies (Santa Clara, CA, USA). Each sample was tested 3 times, and as the relative error was less than 1%, the average value was taken as the final result.

#### 2.2.6. ^27^Al Magic-Angle Spinning NMR

The samples were characterized by Bruker 400M NMR spectrometer produced by Bruker Technology Co., Ltd. (Billerica, MA, USA) Each sample was screened through a 200-mesh sieve, and the angle between the rotation axis and the magnetic field was set to β = θ = 54.7°. Then, ^27^Al spectroscopy was performed on each sample.

#### 2.2.7. TEM

The microscopic morphology and structure of the samples were analyzed using the JEOL JEM 2100F (JEOL Ltd., Tokyo, Japan) transmission electron microscope. The operating voltage of this microscope was set at 200 KV, with an electron wavelength of 0.0251 Å and a resolution of 0.23 nm. The samples were first dispersed in water and then underwent ultrasonic dispersion for 30 min. Subsequently, the water-dispersed droplets of the samples were placed onto a copper mesh containing a carbon-supported film. After natural drying, the samples were subjected to testing.

#### 2.2.8. Viscosity

According to GB/T 5005-2010 drilling fluid material specification, montmorillonite colloid test samples with different interlayer metal cations were prepared, and the samples were tested by NDJ-8S digital rotary viscometer manufactured by Shanghai Jingtian Electronic Instrument Co., Ltd. (Shanghai, China). Each sample was tested 3 times; the relative error was less than 2%.

#### 2.2.9. Swelling Capacity

The swelling capacity of each sample was tested by the method described in JC/T 2059-2011. Each sample was tested 3 times; the relative error was less than 1%.

## 3. Results

### 3.1. Basic Structure of Montmorillonite and Its Gel Properties

All the five selected montmorillonite samples were characterized by XRD, PSD, zeta potential, viscosity and swelling capacity, and the test data are listed in Table 2.

As can be seen from Table 2, there is a significant correlation between the thickness in the 001 crystal plane direction D_001_, the particle size D90, and zeta potential of montmorillonite and its gel properties. The smaller the D_001_ and the D90 value, the greater the zeta potential of montmorillonite, the greater its viscosity and swelling capacity. In addition, there was no clear correlation between the d_001_ value of each sample and its gel properties.

The thickness in the 001 crystal plane direction and particle size of montmorillonite is a manifestation of its lamellar accumulation degree. Small thickness and particle size indicate that the lamellar accumulation is less and loose, and easy to peel off. Zeta potential is an important indicator of the stability of colloidal systems, and the higher the Zeta potential, the easier it is to form a stable colloidal system. The increase in the swelling capacity of montmorillonite often leads to an increase in its viscosity [10]. When the montmorillonite absorbs water and expands, the crystal layer spacing expands, the particle volume increases and the contact between them becomes closer. From a microscopic perspective, the expanded montmorillonite particles can better connect with each other in the dispersed system to form a continuous network structure.

**Figure 1:**
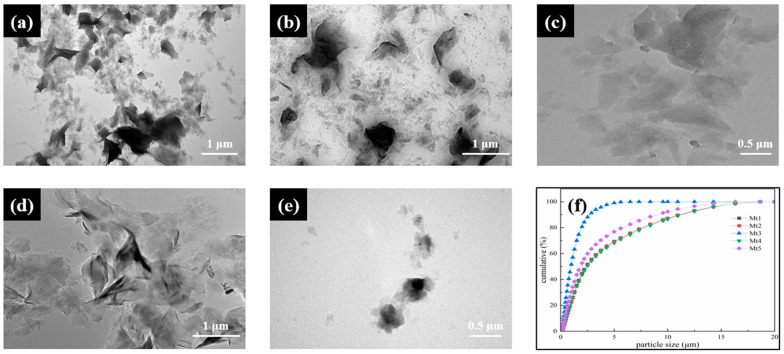
TEM images of (**a**) Mt1, (**b**) Mt2, (**c**) Mt3, (**d**) Mt4, (**e**) Mt5 and particle size distribution of each sample (**f**).

As depicted in Table 2 and Figure 1, of the five samples, Mt3 had the smallest thickness and particle size, the largest zeta potential, while exhibiting the best gel properties. To simplify the discussion, Mt3 will be selected to research the structural regulation of montmorillonite.

### 3.2. Structural Regulation of Montmorillonite by Different Metal Cations

The regulation experiments of different metal cations on the structure of montmorillonite were carried out at room temperature, 25 ± 1 °C. In this research, only the effects of different valence ions on the properties of montmorillonite gel were investigated. Dispersion and ion exchange experiments were carried out using deionized water with pH = 7. In order to ensure complete isomorphism of cations in the interlayer, octahedron, and tetrahedron of montmorillonite, the regulation mechanism of different metal cations on the structure of montmorillonite was studied based on twice the cation exchange capacity of Mt3 (119 mmol/100 g).

An amount of 100 g of Mt3 sample was added to 2000 mL deionized water, hydrated and dispersed for 8 h, and the impurities were removed after full stirring for 10 min. Based on twice the cation exchange capacity of Mt3, chemical reagents containing Li^+^, Na^+^, Mg^2+^, Zn^2+^, Fe^3+^, and Al^3+^ were added for substitution reaction (in this case, the amount of lithium carbonate is 17.61 g, sodium carbonate is 25.23 g, magnesium carbonate basic pentahydrate is 115.19 g, zinc chloride is 32.44 g, ferric chloride hexahydrate is 38.6 g, and crystalline aluminum chloride is 57.48 g.), and the reaction was fully stirred at 60 °C for 2 h. The resulting slurry was centrifuged and separated; the supernatant was collected, was washed with distilled water for 3 times, and was placed in a vacuum drying oven at 60 °C for 24 h to obtain a substituted sample. Then, Mt3-M was obtained, where M is Li^+^, Na^+^, Mg^2+^, Zn^2+^, Fe^3+^, and Al^3+^.

#### 3.2.1. The Effect of Different Metal Cations Regulation on the Interlayer Structure of Montmorillonite

**Figure 2:**
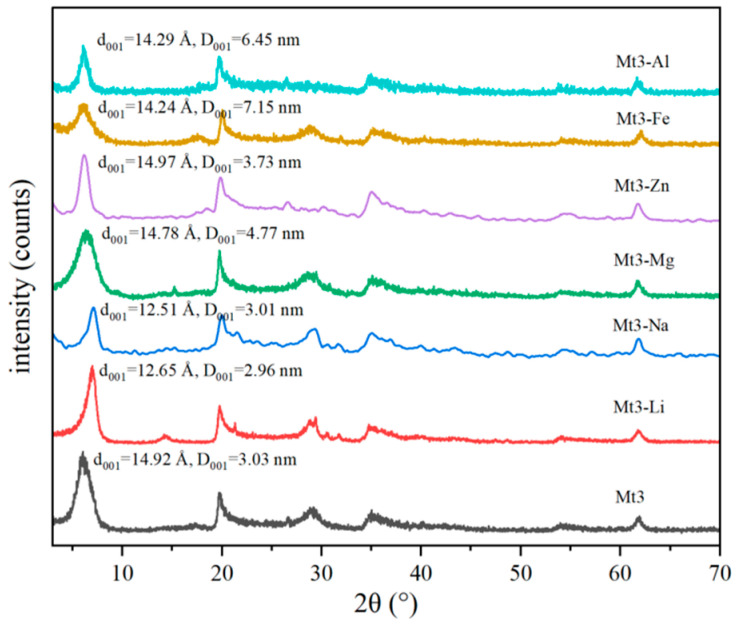
XRD pattern of Mt3 with different substituted cations.

XRD was used to characterize Mt3 regulated by different metal cations, and the influence of interlayer metal ion types on their structure was studied. The results are shown in Figure 2.

As shown in Figure 1, the order of interlayer spacing of Mt3 regulated by different cations is Mt3-Zn > Mt3-Mg > Mt3-Al > Mt3-Fe > Mt3-Li > Mt3-Na. In other words, in Mt3, the order of interlayer spacing regulated by different cations is divalent interlayer cation > trivalent interlayer cation > monovalent interlayer cation [11].

**Figure 3:**
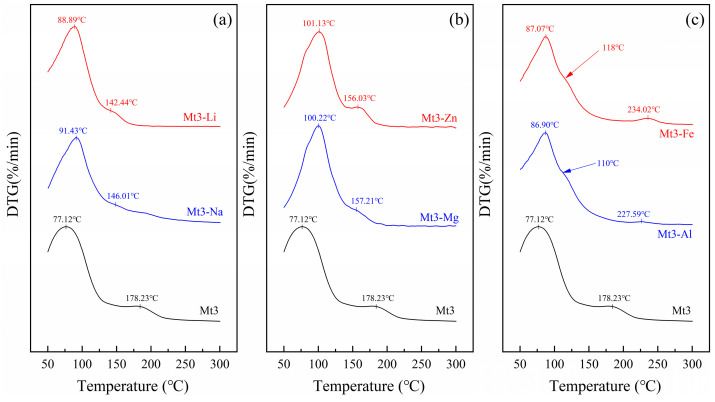
DTG curves at 50–300 °C: (**a**) monovalent cation, (**b**) divalent cation, (**c**) trivalent cation.

In order to further explore the effect of different cations regulation on the interlayer structure of montmorillonite, we analyzed the removal temperatures of water by DTG at 50–300 °C. The results are shown in Figure 3.

The DTG curves showed two endothermic peaks at 50–300 °C. The first peak appears between 50 °C and 105 °C and is caused by the loss of free water. The second appears between 105 °C and 250 °C and may be attributed to the removal of interlayer water from the montmorillonite [12].

After the regulation of polymetallic ions, the removal temperatures of free water and interlayer water of montmorillonite changed significantly. Firstly, as shown in Figure 3, the temperature of the free water increased significantly after ion regulation. Among them, Mt3-Zn and Mt3-Mg increased the most, reaching 101.13 °C and 100.22 °C. Secondly, after the regulation of monovalent and divalent cations, as shown in Figure 3a,b, the temperature of the interlayer water decreased. Mt3-Li and Mt3-Na decreased significantly from 178.23 °C to 142.44 °C and 146.01 °C. Thirdly, after trivalent cation regulation, as shown in Figure 3c, the temperature of the interlayer water is as high as 234.02 °C (Mt3-Fe) and 227.59 °C (Mt3-Al), respectively. But it should be noted that Mt3-Fe and Mt3-Al have a small shoulder peak near 118 °C and 110 °C, respectively (indicated by the arrow in Figure 3c, which should be caused by the bondage of some free water under the strong static electricity of trivalent cations) [13]. In addition, with the exception of Mt2, Mt1, Mt4, and Mt5 also exhibit the above phenomenon.

As the cation valence increases, the electrostatic force becomes stronger. In the process of removing interlayer water, high energy is required to break the hydrogen–oxygen bond and remove the interlayer water from the montmorillonite. When the valence state of cations in the interlayer is the same, the radius of hydrated ions is similar. Ions with greater electrostatic force will cause greater compression between the montmorillonite layers, thereby reducing the layer spacing and reducing the temperature for removing interlayer water. In addition, according to relevant theoretical calculations, Li^+^ and Na^+^ have a layer of bound water between the montmorillonite layers, and the other four ions have two layers of bound water. This is also one of the reasons why the temperature of Mt3-Li and Mt3-Na destructured water is significantly reduced. That is to say, the interlayer spacing and removing interlayer water temperature of montmorillonite are the result of the combined action of hydration ion radius and electrostatic attraction.

#### 3.2.2. Effect of Different Metal Cations Regulation on the Central Ion of Montmorillonite Crystal Structure

According to the method described in Section 2.2.5, the silicon and aluminum contents in the centrifugal supernatant of Mt3 and different cation-substituted samples were determined by ICP. The results are presented in Table 3.

It can be seen from Table 3 that after substitution by different cations, with the exception of the aluminum in the supernatant of the Mg^2+^-substituted sample being the same as Mt3, the content of silicon and aluminum in the supernatant of each sample has changed significantly [14]. This indicates that these cations partially replace silicon and aluminum in the crystal structure of montmorillonite, and the degree of substitution is different [15]. The greater the amount of substitution of the trivalent cation is followed by the monovalent cation, and finally the divalent cation. In the same valence cation, the higher the atomic number, the greater the amount of substitution.

**Figure 4:**
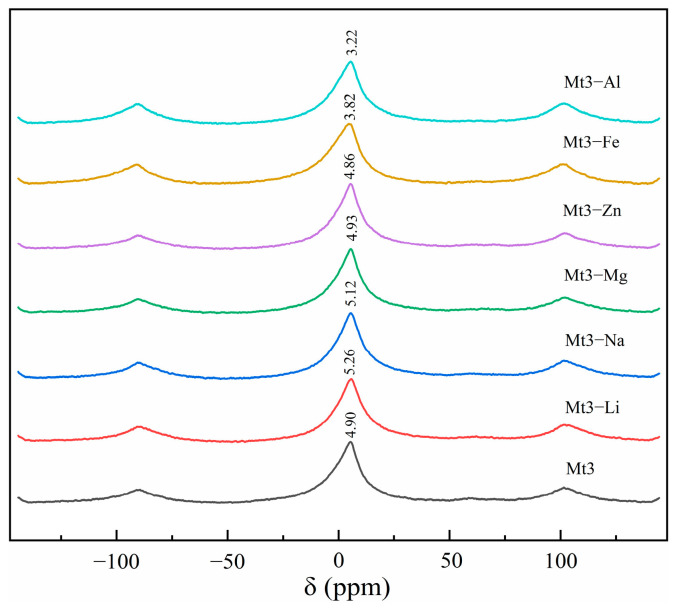
^27^Al solid-state magic-angle NMR spectra for Mt3 and Mt3 regulated by different metal cations.

^27^Al solid-state magic-angle NMR experiments were carried out on Mt3 and Mt3 regulated by different metal cations, as shown in Figure 4, to study the substitution of different metal cations for the central ion of montmorillonite octahedron [16,17].

According to relevant studies, the chemical shift around 3–5 ppm in Figure 4 belongs to hexa-coordinated aluminum. From Figure 4, among different cationic-substituted montmorillonite Mt3, the chemical shifts of trivalent ion-substituted Mt3-Fe and Mt3-Al near 5 ppm shifted most to the high field, by 1.08 ppm and 1.68 ppm, respectively. Fe^3+^ has the same valence as Al^3+^ and a similar ionic radius. Fe^3+^ is more prone to isomorphically replace Al^3+^ in the octahedron. Because of Fe^3+^’s higher electrical properties and field shielding effect compared to Al^3+^, the chemical shifts of hexa-coordinated aluminum move to high fields [18]. The large chemical shift migration of Mt3-Al may result from Al^3+^ substitution in the original vacancies of the Mt3 octahedron, enhancing the field shielding effect. The chemical shifts of divalent ion-substituted Mt3-Mg and Mt3-Zn in hexa-coordinated Al are not prominent. Moreover, compared with divalent ion-substituted montmorillonites, the chemical shifts of six-coordinated aluminum shift slightly to the high field, suggesting that monovalent ions partly substitute octahedral aluminum ions in calcium-based montmorillonite Mt3, with Li^+^ substitution being more evident than Na^+^ [19].

It can be seen that the degree of aluminum substitution by isomorphism in the octahedron is trivalent cation > monovalent cation > divalent cation, which is consistent with the amount of six cations substituting aluminum in the ICP results.

#### 3.2.3. The Influence of Different Metal Cations Regulation on the Octahedral Configuration of Montmorillonite

**Figure 5:**
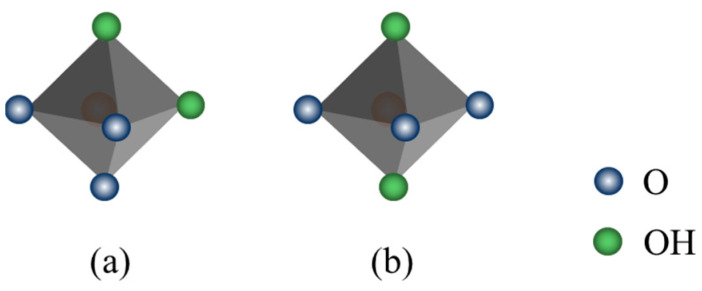
Structure of (**a**) cis-octahedron and (**b**) trans-octahedron of the octahedral configuration of montmorillonite.

Generally, montmorillonite’s octahedron has two configurations regarding hydroxyl group distribution: the cis-octahedron with hydroxyls on the same side and the trans-octahedron with hydroxyls on opposite sides [9], as illustrated in Figure 5.

In dioctahedral montmorillonite, one-third of octahedral positions are vacant. The cis-vacant octahedral sheet is termed the cis-vacancy (cv) configuration, and the trans-vacant one is the trans-vacancy (tv) configuration. The octahedral sheet’s chemical composition and central cation distribution at cis- and trans-checkpoints are crucial for montmorillonite’s octahedral configuration. The tv configuration montmorillonite dehydroxylates in one stage: every two adjacent hydroxyls form a water molecule, leaving an oxygen atom, and the octahedral cation becomes five-coordinate. The cv configuration montmorillonite dehydroxylates in two steps. First, adjacent hydroxyls are replaced by remaining oxygen atoms, making the octahedral cation penta- and hexa-coordinated at cis- and trans-checkpoints, respectively. Second, the cation migrates from the cross-site to the anterior cis-site, needing extra thermal energy. Thus, montmorillonite with the cv configuration has a higher dehydroxylation temperature than that with the tv configuration [20].

**Figure 6:**
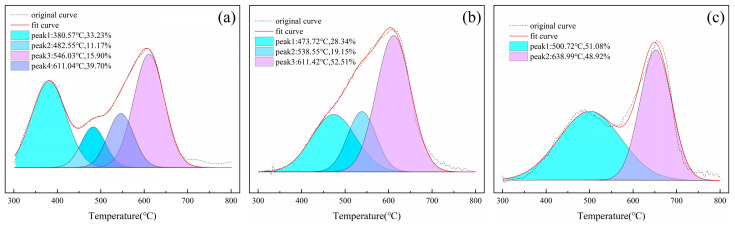

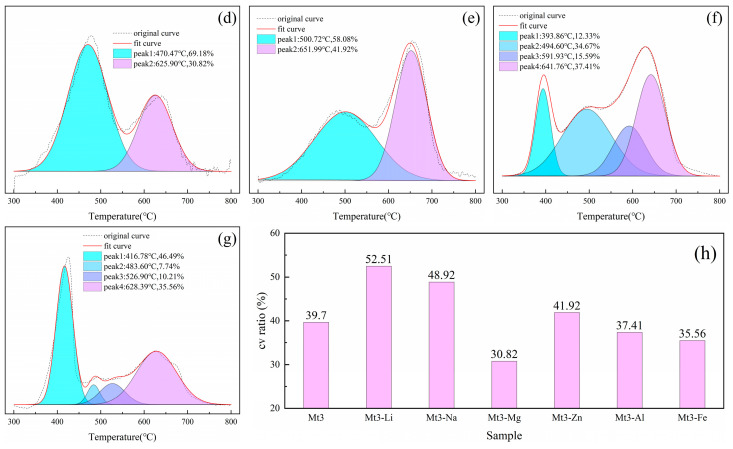
The split peak fitting of the DTG curve (**a**) Mt3, (**b**) Mt3-Li, (**c**) Mt3-Na, (**d**) Mt3-Mg, (**e**) Mt3-Zn, (**f**) Mt3-Al, (**g**) Mt3-Fe, and (**h**) the cv ratio of each sample.

Therefore, thermal analysis is regarded as an effective way to identify the octahedral sheet structure. The dehydroxylation temperature of montmorillonite in the tv configuration is below 600 °C, while that in the cv configuration is above 600 °C. The ratio of peak areas at different temperatures indicates the ratio of cis-to-anti-space configuration. In this research, thermogravimetric analysis (TG) was employed to characterize the montmorillonite octahedral sheet structure. The ratio of the cv configuration was computed by fitting the derivative thermogravimetric (DTG) curve. The split peak fitting of the DTG curve and the cv ratio of each sample are presented in Figure 6.

As shown in Figure 6b,c, after the substitution by monovalent cations, the proportion of the cis-vacancy (cv) configuration in the montmorillonite octahedron increases. As described in Section 3.2.2, monovalent cations can undergo isomorphic substitution with the central ion Al^3+^ in the octahedron. The difference in electronegativity between the monovalent central cation and Al^3+^ makes the octahedron tend to adopt the cis-configuration, resulting in a significant increase in the proportion of the cv configuration. Moreover, the greater the substitution amount of monovalent cations is, the higher the proportion of the cv configuration will be.

Figure 6d depicts that the regulation by Mg^2+^ leads to a significant decrease in the proportion of the cv configuration in the montmorillonite octahedron. As described in Section 3.2.2, Mg^2+^ hardly undergoes isomorphic substitution with Al^3+^ in the montmorillonite octahedron. This may be because, within the octahedron of Mt3, Al^3+^ does not fully occupy all two-thirds of the vacancies. After Mg^2+^ enters, it does not undergo isomorphic substitution with the occupied Al^3+^ but occupies the remaining vacancies. Due to the occupation of Mg^2+^ positions, the dioctahedral structure as a whole transforms towards the trioctahedral structure, manifested as a significant decrease in the proportion of the cv configuration [21].

As shown in Figure 6e, the ionic radius of Zn^2+^ is relatively larger than that of Mg^2+^, making it less likely to occupy the remaining vacancies. Meanwhile, Zn^2+^ undergoes isomorphic substitution with Al^3+^ in the montmorillonite octahedron to a certain extent. The difference in electronegativity makes the octahedron tend to adopt the cis-configuration, and thus the proportion of the cv configuration increases.

However, as illustrated in Figure 6f,g, the radius of Fe^3+^ is similar to that of Al^3+^, so it is easy for Fe^3+^ to undergo isomorphic substitution with Al^3+^. After the substitution, the structural charge of the octahedron remains unchanged. In addition, the Al^3+^ replaced out and the Al^3+^ used for substitution occupy the original vacancies in the Mt3 octahedron, resulting in a decrease in electronegativity. The octahedron tends to adopt the trans-configuration, and the proportion of the cv configuration decreases.

Therefore, the substitution of different cations has a certain impact on the octahedral configuration of montmorillonite. Monovalent cations and divalent cations (except Mg^2+^) have electronegativity differences due to isomorphic displacement, which makes the montmorillonite octahedron tend to a cis-configuration. Although trivalent cations are prone to isomorphic substitution with Al^3+^, after substitution, the charge of the octahedral structure does not change. And because the substitution occupies the original vacancy in the Al^3+^ montmorillonite octahedron, the electronegativity decreases, and the octahedron tends to be in a trans-configuration.

### 3.3. Effects of Different Metal Cations Regulation on the Physical and Chemical Properties of Montmorillonite and Its Gel Properties

#### 3.3.1. Physical and Chemical Properties of Montmorillonite with Different Metal Cations Regulation

**Figure 7:**
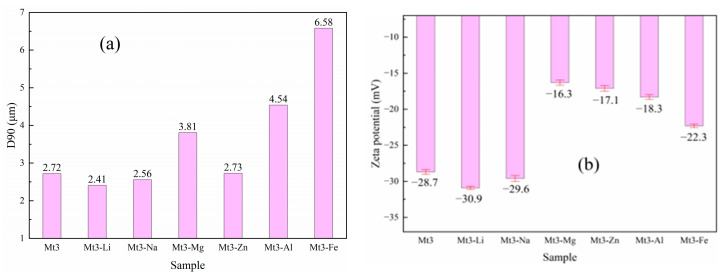
D90 (**a**) and zeta potential value (**b**) of Mt3 with different metal cations regulation.

The changes in D90 and zeta potential of Mt3 after regulated by different metal cations are shown in Figure 7.

As shown in Figure 2 and Figure 7a, different metal cations impact the thickness in the 001 crystal plane direction and particle size of Mt3 differently. Monovalent cations significantly compress montmorillonite’s lamellar spacing, reducing the particle size [22]. Divalent cations notably increase the lamellar spacing, enlarging the particle size. Trivalent cations, though less effective than divalent ones in expanding the lamellar spacing, make particles agglomerate due to their higher electrostatic force, also leading to a significant increase in particle size. This aligns with the results in Section 3.2.1, meaning the effect of different metal cations on Mt3’s particle size results from the combined influence of hydrated ion radius and electrostatic force [23]. Notably, the significant increase in the particle size of Mt3-Mg is likely caused by the sudden change in electrostatic force from Mg^2+^ occupying octahedral vacancies [22].

Figure 7b depicts the effect of different metal cations on the zeta potential of Mt3. The absolute values of the zeta potential of Mt3-Li and Mt3-Na were much higher than that of Mt3, while those of Mt3-Mg and Mt3-Zn were significantly lower. The values for Mt3-Al and Mt3-Fe were between those regulated by monovalent and divalent metal cations. The small amount of isomorphic substitution of monovalent cations and their low electrostatic force result in an increase in the negative charge of the montmorillonite layer [24]. The electric double layer surrounding the particles thickens and overlaps with each other, making the absolute value of the zeta potential of the system larger. Under the regulation of divalent ions, the theoretical charge amount decreases. Due to the small degree of isomorphic substitution and excessive cations, the negative charge of the montmorillonite layer decreases, and the absolute value of the zeta potential also decreases accordingly. After the regulation by trivalent cations, the degree of isomorphic substitution is the maximum, and the negative charge of the montmorillonite layer should increase. However, due to the excessive trivalent cations, the negative charge of the montmorillonite layer decreases sharply. As a result, the absolute value of the zeta potential becomes smaller on the whole, but it is still larger than that under the regulation of divalent ions.

In general, combined with the results of Section 3.2.1, the regulation of the montmorillonite’s surface potential by different metal cations is affected by the degree of isomorphic displacement of cations and their electrical synergy [25]. The order of the impact of cation valence states on the zeta potential of montmorillonite is monovalent cation > trivalent cation > divalent cation. In addition, the thickness in the 001 crystal plane direction, particle size, and zeta potential of Mt1, Mt2, Mt4, and Mt5 all showed the same trend.

#### 3.3.2. Gel Properties of Montmorillonite with Different Metal Cations Regulation

The changes in viscosity and swelling capacity of Mt3 after regulated by different metal cations are shown in Table 4.

As shown in Table 4, different metal cations have a significant impact on the swelling capacity and the suspension viscosity of montmorillonite. The suspension viscosity is Mt-Li > Mt-Na > Mt-Zn > Mt-Mg > Mt-Al > Mt-Fe, except Mt4 and the order of the swelling capacity is Mt-Li > Mt-Na > Mt-Fe > Mt-Al > Mt-Zn > Mt-Mg, except Mt2 and Mt4. Combined with the results of the Section 3.3.1, the suspension viscosity of montmorillonite is negatively correlated with its particle size, and the smaller the particle size, the greater the viscosity. The swelling capacity of montmorillonite is positively correlated with the absolute value of zeta potential. The larger the absolute value of zeta potential, the larger the swelling capacity.

### 3.4. Regulation Mechanism of Different Metal Cations on the Gel Properties of Montmorillonite

As stated above, different metal cations adjust the layer spacing of montmorillonite to regulate its particle structure. Microscopically, this leads to a change in the occurrence state of interlayer water, and macroscopically, it results in a change in gel properties [24]. Moreover, different metal cations regulate the chemical environments and electrical properties through isomorphic substitution of Si^4+^ in the tetrahedron and Al^3+^ in the octahedron of the montmorillonite crystal. Microscopically, this causes a change in the cv ratio of the octahedron structure and macroscopically, it also brings about a change in the zeta potential and gel properties.

**Figure 8:**
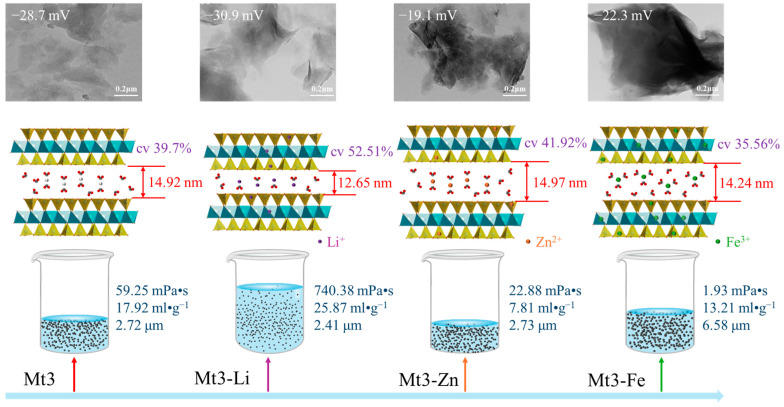
Microscopic and macroscopic behavior of montmorillonite regulated by different metal cations.

In order to describe the regulation process of different metal cations more concisely, Li^+^, Zn^2+^, and Fe^3+^ were selected to represent monovalent, divalent, and trivalent cations, respectively, to analyze the structural regulation of different metal cations on montmorillonite and the mechanism of influence on the gel properties of montmorillonite shown in Figure 8.

As depicted in Figure 8, Mt3-Li exhibits the smallest layer spacing (12.65 nm) and the smallest particle size (D90 = 2.41 μm). In addition, it has the highest cv configuration (52.52%) in octahedron, the largest absolute value of zeta potential (−30.9 mV), as well as the largest viscosity and swelling capacity, which are 740.38 mPa s and 25.87 mL g^−1^, respectively. When monovalent cations regulate montmorillonite, their smaller hydrated ion radius and electrostatic force significantly decrease the layer spacing of montmorillonite. However, their isomorphism displacement leads to a notable increase in the proportion of cv configuration by changing the electronegativity of the octahedron. Overall, the particle size is significantly reduced and the absolute value of the zeta potential is significantly increased. It is easy for it to peel off and expand in water to form a uniform and stable colloidal substance, possessing the maximum viscosity and expansion volume. In other words, it has the best gel performance [26].

Mt3-Zn exhibited the largest layer spacing (14.97 nm) and a relatively larger particle size (D90 = 2.73 μm). In addition, it has a decreased proportion of cv configuration (41.92%) in octahedron, the smallest absolute value of zeta potential (−19.1 mV), a viscosity of 22.88 mPa s, and the smallest swelling capacity (7.81 mL g^−1^). When divalent cations regulate montmorillonite, the combined effect of the hydrated ion radius and electrostatic force causes a significant increase in the layer spacing. Although the degree of isomorphic displacement of divalent cations is small, they affect the proportion of the cv configuration by occupying the original vacancies in the octahedron. Overall, this results in a smaller particle size and a lower absolute value of the zeta potential. It is not easy for it to peel off and expand in water. The swelling capacity is significantly reduced, yet it still has a certain viscosity and can form relatively stable colloidal substances.

The layer spacing of Mt3-Fe is 14.24 nm, and it has the largest particle size (D90 = 6.58 μm). The cv configuration of its octahedron is the lowest (35.56%), with a zeta potential of −22.3 mV. Its viscosity is the smallest (1.93 mPa s), and the swelling capacity is 13.21 mL g^−1^. When the trivalent cation regulates montmorillonite, its large hydrated ion radius and electrostatic force limit the expansion of the layer spacing. Although the degree of isomorphic displacement is the largest, the Al^3+^ replaced from the octahedron reduces the electronegativity as it occupies the original vacancy of the octahedron, which leads to a decrease in the proportion of the cv configuration. Overall, the performance is characterized by a larger particle size and a significantly reduced viscosity. However, due to its relatively larger absolute value of the zeta potential, the montmorillonite regulated by the trivalent cation still has a certain swelling capacity in water.

Therefore, the effect of different metal cations on the particle size of montmorillonite is the result of the combined effect of hydrated ion radius and electrostatic power, while the regulation of different metal cations on the surface potential of montmorillonite is influenced by the degree of isomorphism displacement of cations and the synergistic effect of cation itself. Metal cations in different valence states affect the interlayer structure and crystal structure of montmorillonite in different forms, resulting in changes in its gel properties.

## 4. Conclusions

In this study, through analyzing the relationship between the basic structures of five kinds of montmorillonite and their gel properties, the Mt3 samples with the best gel properties were selected for the regulation by different valence metal cations. It was found that the regulation of different metal cations has a significant impact on the interlayer structure and crystal structure of montmorillonite. Microscopically, this is manifested as the changes in the state of the interlayer water and the cv configuration ratio of the octahedral structure. Macroscopically, it is manifested as the changes in gel properties resulting from the variations in particle size and zeta potential. The following conclusions are drawn from this study:

(1) The suspension viscosity of montmorillonite is negatively correlated with its particle size (the smaller the particle size, the greater the viscosity). The impact of different metal cations on montmorillonite’s particle size results from the combined effect of hydrated ion radius and electrostatic force. The order of montmorillonite particle size regulated by different cations is trivalent cation > divalent cation > monovalent cation.

(2) The swelling capacity of montmorillonite is positively correlated with its absolute value of zeta potential (the larger the absolute value of zeta potential, the larger the swelling capacity). The regulation of different metal cations on the surface potential of montmorillonite is influenced by the degree of isomorphism displacement of cations and the synergistic effect of cation itself. The order of the zeta potential of montmorillonite regulated by different cations is monovalent cation > trivalent cation > divalent cation.

(3) When monovalent cations regulate montmorillonite, their smaller hydrated ion radius and electrostatic force significantly decrease the layer spacing of montmorillonite. However, their isomorphism displacement leads to a notable increase in the proportion of cv configuration by changing the electronegativity of the octahedron. Overall, the particle size is significantly reduced and the absolute value of the zeta potential is significantly increased. It is easy for it to peel off and expand in water to form a uniform and stable colloidal substance, possessing the maximum viscosity and expansion volume. In other words, it has the best gel performance.

## Figures and Tables

**Table 1:** Five kinds of raw montmorillonite sample details.

Sample	Place of Origin	Mineral Composition/%
Mt1	Qingfengshan, Liaoning	Montmorillonite 95.41, Quartz 4.59
Mt2	Shahai, Liaoning	Montmorillonite 94.59, Calcite 5.41
Mt3	Xuanhua, Hebei	Montmorillonite 99, Quartz 1
Mt4	Zhangjiakou, Hebei	Montmorillonite 91.24, Quartz 8.76
Mt5	Chifeng, Inner Mongolia	Montmorillonite 96.4, Quartz 3.6

**Table 2:** Basic properties and gel properties of montmorillonites.

Sample	d_001_/Å	D_001_/nm	D90/μm	Zeta Potential/mV	Viscosity/mPa·s	Swelling Capacity/mL g^−1^
Mt1	15.33	8.29	11.08	−17.6	1.6	5.02
Mt2	14.96	8.56	11.21	−16.3	8.3	7.87
Mt3	14.92	3.03	2.72	−28.7	59.2	17.93
Mt4	15.12	8.94	11.33	−21.7	11.9	6.55
Mt5	15.48	5.50	8.99	−25.1	53.7	14.60

**Table 3:** Silicon and aluminum content in supernatants of each sample.

Concentration	Sample						
	Mt3	Mt3-Li	Mt3-Na	Mt3-Mg	Mt3-Zn	Mt3-Fe	Mt3-Al
Si/ppm	0.83	2.44	2.10	2.32	3.37	9.79	7.43
Al/ppm	0.12	1.32	0.88	0.12	0.23	9.64	233.61

**Table 4:** Viscosity and swelling capacity of montmorillonite with different metal cations regulation.

Sample	Mt1	Mt1-Li	Mt1-Na	Mt1-Mg	Mt1-Zn	Mt1-Al	Mt1-Fe
Viscosity/mPa·s	1.6	267.23	255.71	-	1.32	-	1.13
Swelling capacity/mL·g^−1^	5.02	11.33	10.04	2.33	4.92	5.05	6.37
Sample	Mt2	Mt2-Li	Mt2-Na	Mt2-Mg	Mt2-Zn	Mt2-Al	Mt2-Fe
Viscosity/mPa·s	8.3	341.7	301.27	2.6	6.9	1.41	1.62
Swelling capacity/mL·g^−1^	7.87	14.52	12.56	5.58	4.32	6.01	7.22
Sample	Mt3	Mt3-Li	Mt3-Na	Mt3-Mg	Mt3-Zn	Mt3-Al	Mt3-Fe
Viscosity/mPa·s	59.25	740.38	680.01	8.14	22.88	2.04	1.93
Swelling capacity/mL·g^−1^	17.93	25.87	21.30	6.99	7.81	11.05	13.21
Sample	Mt4	Mt4-Li	Mt4-Na	Mt4-Mg	Mt4-Zn	Mt4-Al	Mt4-Fe
Viscosity/mPa·s	11.9	372.17	377.33	4.33	7.2	2.11	1.81
Swelling capacity/mL·g^−1^	6.55	15.33	15.46	3.35	4.22	5.38	6.09
Sample	Mt5	Mt5-Li	Mt5-Na	Mt5-Mg	Mt5-Zn	Mt5-Al	Mt5-Fe
Viscosity/mPa·s	53.7	701.7	669.19	7.72	19.38	2.06	1.87
Swelling capacity/mL·g^−1^	14.6	26.03	25.36	5.9	6.03	9.27	10.31

## Data Availability

The original contributions presented in this study are included in the article. Further inquiries can be directed to the corresponding authors.
